# Aquatic Alchemy

**DOI:** 10.1289/ehp.113-a110

**Published:** 2005-02

**Authors:** Lance Frazer

The average Earthbound American, estimates the U.S. Environmental Protection Agency (EPA) uses nearly 24 gallons of water daily for personal uses such as drinking, toilet flushing, hygiene, and cleaning. International Space Station (ISS) crew members, on the other hand, are limited to little more than half a gallon per day. Yet, with that little bit of water weighing about 5 pounds, and the cost of lifting something into low Earth orbit can top $10,000 per pound, it would make far better economic sense to provide astronauts with water on-site.

Aerospace engineers are busy working on a modern-day form of alchemy, finding new ways to find water in space without having to lug it along from Earth. And while many of the technologies are being developed with an eye toward the stars, certain aspects may be suitable for use here at home on planet Earth.

## Pulling Water from the Air

Every time an astronaut exhales, washes up, or urinates, water is involved. In an effort to minimize the amount of fluid lifted into orbit, the National Aeronautics and Space Administration (NASA) is seeking ways to recapture that water, clean it, and store it for reuse.

The ISS currently provides clean water through the use of a water recovery system that reclaims wastewater such as used oral hygiene water, urine, and cabin humidity condensate. But the space environment offers unique challenges to the provision of water. For example, water must contain no dissolved gases, as gas doesn’t separate well at zero gravity, either in tanks or in the body. Therefore, the water must be purified to a level exceeding Environmental Protection Agency standards for Earth drinking water. The WRS consists of a urine processor assembly (essentially a still that boils off water, leaving a thick waste layer behind) and a water processor assembly developed by researchers at NASA’s Marshall Space Flight Center and Hamilton Sundstrand Space Systems International.

According to Dave Parker, program manager for Hamilton Sundstrand’s water processor program, the water processor assembly is a multistage system that uses filters to remove particulate matter and salts. The water is then run through a catalytic oxidizer to remove low-molecular-weight organic molecules such as alcohols. This part of the process takes place at a temperature of 275°F, and under pressure so it doesn’t flash to steam, Parker explains. The system then removes any byproducts and remaining dissolved gasses, and next goes through an ion exchange process to remove the oxidation products.

This process, according to Parker, can produce about 1.5 gallons of treated water per hour, and uses approximately 700 watts of power. To reduce maintenance times and the volume of consumables that must be delivered from Earth, the system has been designed with an 80- to 90-day change-out schedule for particulate filters, and a 60- to 70-day schedule for chemical filters, with no more than 12 hours of maintenance time required per year.

The water processor assembly is designed to provide limited amounts of highly purified water with minimal energy consumption and maintenance, but Parker believes the system could be scaled up for Earth usage. “We’re producing as nearly pure potable water as you’re likely to find anywhere,” he says. “The question you’d have to answer on Earth is whether you need water of that purity.” Parker suggests the system could be used in military applications, to protect crews against chemical or biological attack, or aboard naval vessels. It could also work in hospitals, where high-quality water is important. “We’re also working with the Army to design a system to create potable water from diesel exhaust,” he says.

There are also substances often encountered in terrestrial water that you’ll never find aboard the space station, such as arsenic, mercury, and heavy metals, but Parker says the water processor assembly could be adjusted to deal with these substances in a limited-application water stream. “While you could plug this system into a municipal water system, I suspect that the economics wouldn’t work. The system operates to higher standards, and avoids things that municipalities traditionally employ, such as the addition of chlorine to sterilize water. NASA doesn’t allow any chlorine aboard the ISS, so we use heat instead. That wouldn’t be economical in a multimillion-gallon-throughput municipal system.”

## Further Out, Longer Stay

With NASA looking in more detail at a manned Mars mission—which would involve 40 million miles and 3–5 years—work has begun on a fully regenerative water recycling system, one that can provide a crew with adequate water for drinking and hygienic needs for up to three years without recharging. Enter the Vapor Phase Catalytic Ammonia Removal (VPCAR) system.

Michael Flynn, the project’s principal investigator at NASA’s Ames Research Center, says VPCAR has been designed to mimic the natural hydrologic cycle. “On Earth, you open the tap, drink water, produce waste, treat the waste, and discharge it back into the ocean,” he explains. “The sun heats the water, which evaporates and forms clouds. Those clouds are exposed to ultraviolet radiation, which destroys organic contaminants, and then rain falls to begin the cycle again. We’ve integrated all of these processes into a single small machine. We take in waste water, vaporize it, oxidize organic contaminants, re-condense it, and the water is ready for use.”

In general, says Flynn, nonregenerative technologies (like the water processor assembly), are dominated by adsorptive technologies such as activated carbon, which boast low power consumption. “You make the trade-off of having a shuttle fly up every ninety days to resupply expendables like filters because the system uses relatively little power,” he says. On the other hand, there will be no resupply opportunity on a manned Mars mission, so it’s desirable to spend more on power than on resupply. For example, Flynn says, the nonregenerative systems aboard the ISS only use an estimated 123 watt hours/kg, compared to what he says is around 300 watt hours/kg for the fully regenerative VPCAR.

VPCAR works by sending the waste stream across a wiped-film rotating disk evaporator, which removes inorganic salts and nonvolatile large-molecular-weight organic contaminants. Lightweight organic molecules and ammonia, which are volatilized in the evaporator, are oxidized by a high-temperature catalytic oxidation reactor, converting these organics into carbon dioxide, water, and nitrous oxide. This high-temperature process also helps destroy any biologically active organisms in the waste stream.

Full characterization studies of VPCAR have been completed, and the system, Flynn says, meets all NASA specifications. The next step will be a test aboard training aircraft, followed by full-scale flight-testing during a proposed lunar mission.

VPCAR, Flynn admits, would have limited applications on Earth, although some aspects of it are being considered, including the evaporative portion of the system, which has been examined by the U.S. South Pole research station, which wants to purify its sewage such that it could be used to make an ice layer for the research station’s runway. “We also have some rural Alaskan tanneries looking at using the system to recycle their waste, and some oil companies are looking at it as a technology to separate oil and water,” Flynn says. “VPCAR is also being looked at as an alternative method of distilling salt water into fresh.”

## Further and Longer: Bearing ARMS

While some researchers are trying to make sure there are no bacteria in the water, others are going out of their way to welcome them. Tony Rector is a bioprocess engineer with Dynamac Corporation at Kennedy Space Center, where he and colleagues are working on a project called ARMS—the Aerobic Rotational Membrane System.

ARMS consists of a clear Plexiglas reactor vessel, filled with 115 tubes (dubbed “membranes”) that are home to a community of bacteria. Oxygen moves from the inside of each membrane to its outside surface, where bacteria are present in colonies called biofilms. Contaminated water flows past these biofilms, where the bacteria can use the oxygen to transform undesirable compounds found in the wastewater to less harmful compounds. Biological treatment reactors using membranes aren’t new technology, but this system is innovative in that the membranes rotate, exposing more of the bacteria to more of the infused oxygen and the contaminants that provide their nourishment.

“Biological systems like these can achieve high treatment efficiencies with low mass and energy requirements,” says Rector. “By rotating the membranes, we can enhance mass transfer, making the system as efficient as possible.”

As living organisms, bacteria are subject to many of the same stresses that will impact human astronauts, and if the bio-community goes down due to some shock (for example, an unexpected radiation dose or loss of heat or oxygen) it can be days before the community can be resuscitated. “Part of our long-term plan is to subject these organisms to a variety of shocks, and see how they react and recover from such events,” says Rector. “We’d also like more information on their ideal living conditions, what bacterial species are most tolerant and resilient, and other information of that sort.” Rector’s research group will also be examining different biochemical processes in a variety of bacterial species, including nitrification bacteria and hydrogen-oxidizing bacteria. Potential candidate bacteria will have to be evaluated for their tolerance for pharmaceuticals, hormones, antibiotics, and other substances that may be excreted by the astronauts using the system.

Because of the potential sensitivity of the living organisms, and because bacteria are unable to process all of the materials in a typical waste stream, Rector envisions using ARMS as a first phase in an overall water treatment process in space, followed by a chemical/mechanical process. “Our goal is to reduce the contaminant loading on these processes and therefore reduce the size and energy requirements for larger-scale physical and chemical systems,” Rector says.

“Bioregenerative research, focusing on water recovery, has been conducted at NASA research centers for many years. While we are in the initial stages of the ARMS project, we are very encouraged by its current performance,” says Rector. “Depending on mission scenarios and timelines, it could be five or six years before we can complete our investigation of the biological and mechanical component aspects of this system. I think there’s a lot of potential, but there’s still a lot of research to be done.”

ARMS, he says, could well find maritime applications, perhaps aboard cruise ships, which must thoroughly treat their waste before discharge. “This system will find its best use in a small environment,” he says.

“Best use in a small environment” seems to be a key descriptor of all of these processes. None of the individuals involved in research on the three systems envisions them as replacements for large-scale municipal water treatment facilities, but all agree they should work—and work well—in a confined environment, where it’s necessary to have very clean water, and all that’s available as feedstock is what Flynn describes as “the nastiest stuff imaginable.” The military, medical facilities, any place with a need for high-quality water supplies where conventional sources are unreliable—all are potential down-to-Earth targets for NASA’s spaceborne water systems.

## Figures and Tables

**Figure:**
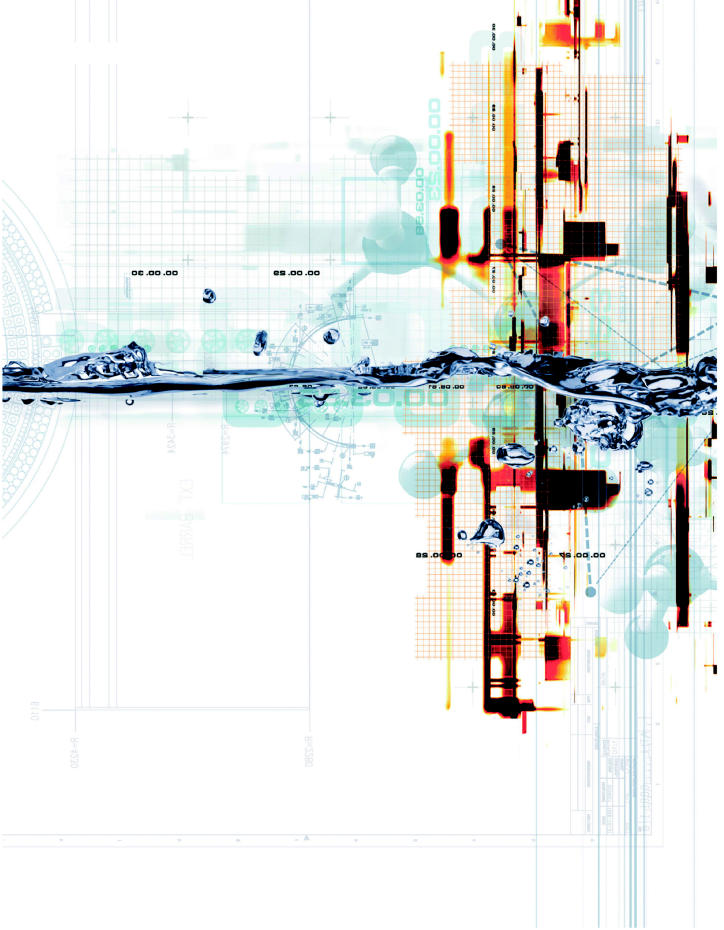


**Figure:**
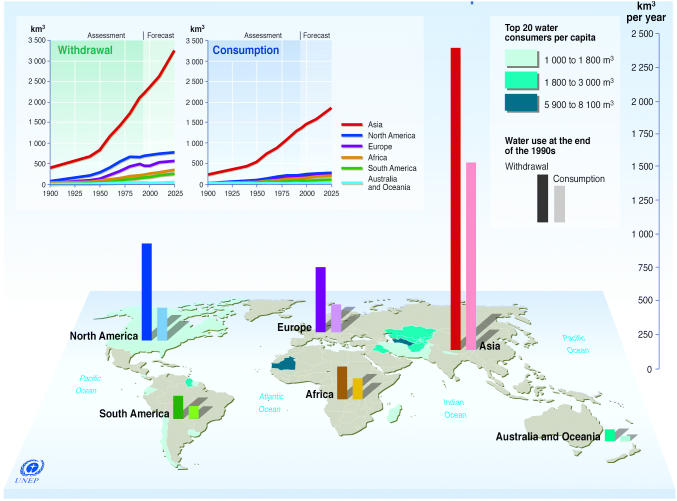
**Sources.** Shiklomanov IA, State Hydrological Institute, United Nations Educational, Scientific, and Cultural Organisation. 1999. World Resources 2000–2001—People and Ecosystems: The Fraying Web of Life. Washington, D.C.: World Resources Institute; Harrison P, Pearce F. AAAS Atlas of Population 2001. Berkeley, CA: American Association for the Advancement of Science, University of California Press.

**Figure:**
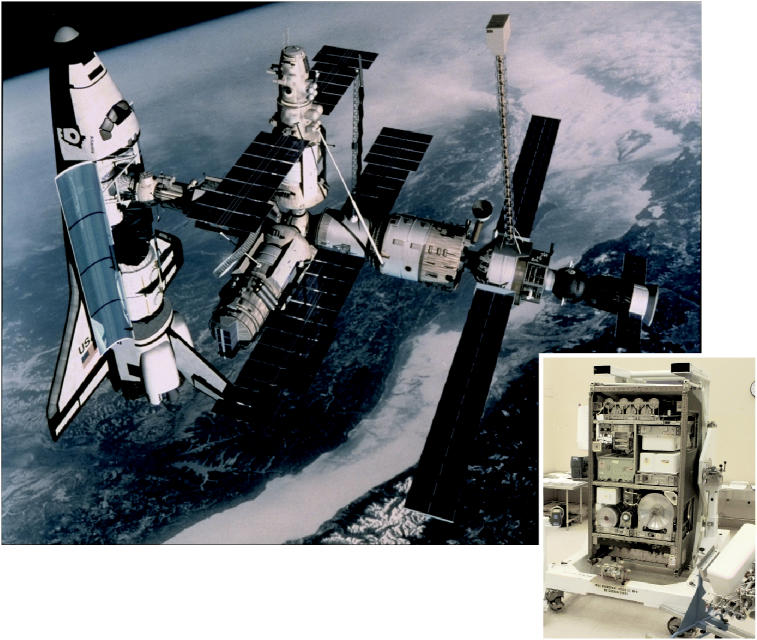
** Precious cargo.** The International Space Station and (inset) the water recovery system developed by Hamilton Sundstrand that’s aboard.

**Figure:**
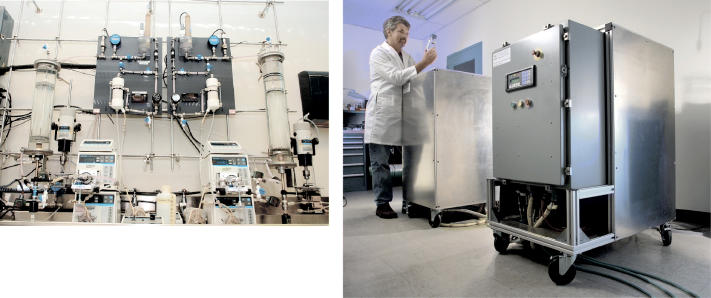
**Space drinks.** (left) The photo shows two ARMS reactors which are currently being evaluated. It shows the teststand for the ARMS systems, which includes the reactor vessel and sensors. (right) VPCAR being tested in the lab.
